# Multilayer Super-Translucent Zirconia for Chairside Fabrication of a Monolithic Posterior Crown

**DOI:** 10.1155/2022/4474227

**Published:** 2022-03-23

**Authors:** Sven Rinke, Anna Metzger, Holger Ziebolz

**Affiliations:** ^1^Dental Clinic Prof. Dr. S. Rinke, Dr. M. Jablonski & Dr. H. Ziebolz, Hanau, Germany; ^2^University Medical Center Göttingen, Department of Prosthodontics, Göttingen, Germany

## Abstract

This case report describes the chairside fabrication of a monolithic posterior crown using a multilayer super-translucent zirconia material. According to the manufacturer's information, the newly introduced multilayer zirconia (4-YTZP) offers a unique combination of fracture strength (>850 MPa with speed-sintering) and improved optical properties, thus allowing a reduced minimum material thickness and optional temporary luting. By using up-to-date components of the CEREC system, including superfast dry-milling and a speed-sintering process, the fabrication of a monolithic zirconia crown is possible within an acceptable timeframe for the chairside workflow (60-75 min). The usage of a multilayer super-translucent material allows for the individualization of the restoration, typically in a single combined stain and glaze firing. However, it should be noted that clinical data for this type of restoration are still sparse. Therefore, manufacturer recommendations regarding indication, preparation, and cementation must be followed very strictly.

## 1. Introduction

Since the introduction of translucent zirconia ceramic materials more than 10 years ago, these materials allow the fabrication of monolithic crowns and fixed partial dentures (FPDs) [1, 2].

**Figure 1:**
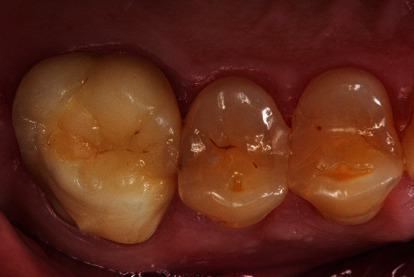
Zirconia materials of the first generation could only be used for veneered restorations, especially the molar region was prone to an increased risk for fractures of the veneering ceramics.

Omission of ceramic veneering reduces the risk of fractures in the veneering ceramics to a great extent (Figure 1). Existing clinical data show a significantly reduced technical complication rate in the posterior region in comparison to veneered restorations made of zirconia [3–5]. So far, the use of zirconia ceramic materials for the chairside fabrication of monolithic crowns and FPDs was not considered reasonable due to the comparatively long sintering times [6, 7]. The development of so-called speed-sintering processes in combination with translucent zirconia ceramic materials now offers the option of a chairside processing of zirconia crowns and FPDs [1, 6, 8, 9]. However, the advantage of zirconia materials in the chairside workflow and the choice of materials should be discussed in general [1, 10, 11].

High-strength glass-ceramics are the most frequently used materials in chairside processing (e.g., IPS e.max CAD, Ivoclar Vivadent, Schaan, Liechtenstein; CERECTessera, Dentsply Sirona, Charlotte, USA) [7, 11–14]. Their 3-point flexural strength of more than 450 MPa is sufficient for the fabrication of single crowns and 3-unit FPDs up to the first premolar, provided that the following preconditions are considered: The minimum material thickness required for the restoration is 1.0 mm, the adhesive luting is indispensable, and temporary cementation is not possible [15, 16].

High translucency that allows for a distinct chameleon effect and good conditioning properties for adhesive luting is the main advantage of this group of materials [12, 17]. The present clinical data document high success and survival rates for chairside-fabricated single-tooth restorations over observational periods of up to 10 years [10, 12, 18, 19].

Meanwhile, translucent zirconia ceramic materials are available with different modifications; the translucency is mainly caused by varying percentages of alumina and yttria. Although the first generation of zirconia with a portion of 3 mol% yttrium oxide shows high 3-point flexural strength (ISO 6872-2015) in the region of 1,000 to 1,200 MPa, the translucency of this group of materials is relatively low compared to glass-ceramic materials [1]. By reducing the alumina share, an increased translucency could be achieved in zirconia materials of the second generation, leading to nearly unchanged mechanical properties [20]. For monolithic crowns and FPDs of the second generation, first clinical studies document a significant reduction in the technical complication rate of tooth- and implant-supported single-tooth restorations in comparison to veneered zirconia restorations [3, 4, 10, 21].

Zirconia materials of the third generation contain 5 mol% yttrium. This allows a translucency comparable with that of high-strength glass-ceramic materials. On the other hand, in comparison with classic zirconia materials with an yttria content of 3 mol%, these materials show a reduced strength [2, 10, 20]. Therefore, these zirconia materials do not offer a wider range of indication in comparison to high-strength glass-ceramics. They are suitable for single crown restorations and 3-unit FPDs up to the first premolar [1, 10].

**Figure 2:**
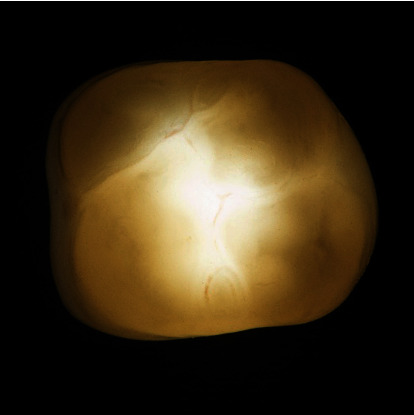
Transillumination of a zirconia crown of the fourth generation (CEREC MTL Zirconia, Dentsply Sirona).

The fourth generation of zirconia materials contains 4 mol% yttrium. Although this leads to a slight reduction in translucency compared to high-strength glass-ceramics, the mean flexural strength increases to 850–1,000 MPa [1, 2, 20–22] (Figure 2).

This modification results in 3 clinically relevant aspects: First of all, according to the manufacturers' information, these materials are suitable for the fabrication of anterior and molar single crowns and 3-unit FPDs, allowing for a restoration with FPDs to replace the first or second molar. Additionally, due to the increased strength of the material, the minimum material thickness can be reduced to 0.6–0.7 mm. The third clinically relevant advantage is the approval of this material group for temporary cementation [2, 20].

Summarizing these material properties, it is apparent that especially zirconia materials of the fourth generation are a meaningful addition to high-strength glass-ceramics in the chairside workflow, as they allow for a wider range of indications while offering a more minimal-invasive preparation.

In this combination, the main indications for the use of high-strength glass-ceramics are inlays, onlays, partial crowns, crowns, and veneers. Zirconia materials of the fourth generation should preferably be used for the fabrication of single crowns and 3-unit FPDs [1]. For the every-day chairside processing of zirconia materials in a dental practice, the processing time should be shortened to a single appointment. In this context, the following aspects are important:

- *Short Processing Times and Dry Processing to Avoid Drying Prior to Sintering*. As zirconia materials are processed in a presintered, chalk-like state, processing times of 5-6 minutes for a single crown can be achieved when using an appropriate milling unit (e.g., Primemill, Dentsply Sirona, Charlotte, USA) [23].
- The sintering period should be kept as short as possible. By choosing a speed-sintering program, it can be reduced to less than 20 minutes. In vitro studies show that speed-sintering does not have a negative influence on the material properties (hydrothermal aging behavior, Weibull characteristics, hardness, and translucency) of appropriate zirconia materials [8, 9, 24]

Ideally, individualization of the restoration should be finished in a single stain and glaze process. This can be achieved by using multilayer zirconia blocks. Pigmented layers of varying intensities simulate the typical color gradient of a natural tooth. These multilayer zirconia blocks consist of 4–5 color layers in different pigmentations while showing similar translucencies [21]. In comparison with a single-color block, this integrated color gradient simplifies the individual characterization of the restoration significantly. In ideal circumstances, a multilayer material requires only one combined stain and glaze process to achieve an esthetically perfect result [25, 26].

Until now, the mechanical and optical properties, as well as the abrasion and fatigue behavior of multilayer translucent zirconia materials, have been evaluated in several in vitro studies [20, 26–28]. However, reports on clinical experiences are limited to case studies and reports with labside-fabricated restorations with observational periods up to 24 months [29, 30]. Clinical data for chairside-fabricated restorations from multilayer translucent zirconia materials are still sparse [23].

The present case report demonstrates the chairside fabrication of a monolithic single-tooth restoration by using a new multilayer zirconia material of the fourth generation (CEREC MTL Zirconia, Dentsply Sirona, Charlotte, USA) with the current version of the CEREC system (Primescan&Primemill, Dentsply Sirona, Charlotte, USA).

## 2. Case Presentation

**Figure 3:**
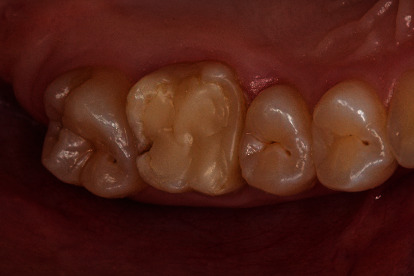
Initial situation: endodontically treated first upper molar with an insufficient composite restoration.

During a prophylaxis appointment, an insufficient composite restoration of an endodontically treated first upper molar was detected in a 31-year-old female patient (Figure 3). X-ray diagnostics revealed a sufficient endodontic treatment ad-apex without apical findings, thus allowing for a prosthodontic restoration.

After removing the existing restoration, the tooth was prepared and treated with an adhesive build-up (Clearfil DC core plus, Dentin, Kuraray Noritake Dental Inc., Tokyo, Japan).

**Figure 4:**
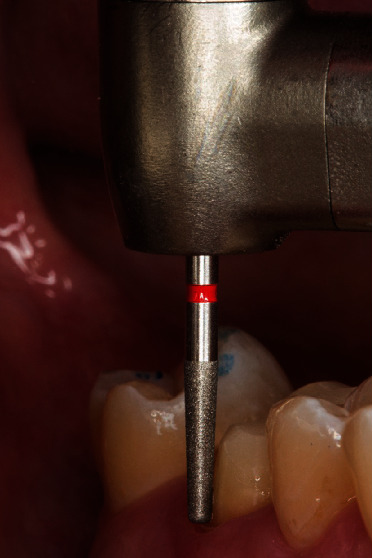
Design of the preparation limit: shoulder with rounded inner angle (8951.314.017, Komet Dental, Lemgo, Germany).

**Figure 5:**
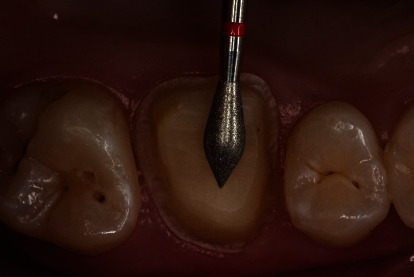
Preparation of the occlusal surface (8899.314.027, Komet Dental, Lemgo, Germany).

The equigingival preparation limit was performed as a shoulder with rounded inner angle (Figure 4). The occlusal design was prepared with a rhombic instrument to achieve an aperture angle of 120–140° (Figure 5) [31, 32]. The axioocclusal line angles were rounded with the same instrument. The occlusal reduction was approximately 1 mm, and the cutting depth at the preparation limit was ranging from 0.8 to 1 mm. Thus, a minimum occlusal and axial material thickness of 0.8 mm was ensured.

**Figure 6:**
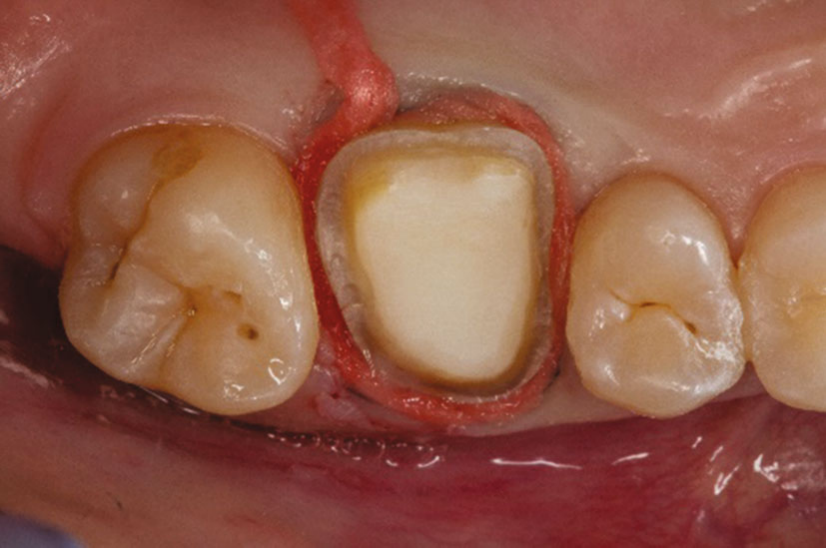
Preparation for the intraoral scan. For increased lateral extrusion of the soft tissues, a layer of cotton is applied after a knitted retraction cord is placed. The cotton is removed immediately prior to oral scanning.

Before digital impression taking, a retraction cord (Ultrapak Clean Cut, size 1, Ultradent Products, South Jordan, USA) was applied. In order to achieve maximum lateral soft tissue displacement, one layer of hemostatic cotton was placed as top layer (Figure 6). In addition, for a sufficient hemostasis and conditioning of the soft tissues, a compression cap (Roeko Comprecap, size 5, ColteneWhaledent, Cuyahoga Falls, USA) was applied. The patient was instructed to fix the cap by biting down. After a residence time of 10 minutes, the digital impression was taken (Table 1).

Immediately before intraoral scanning (CERECPrimescan AC, Dentsply Sirona, Charlotte, USA) of the preparation quadrant, the compression cap and hemostatic cotton were removed and the scanning area was gently air-dried. Throughout the scanning process, the retraction cord remained in situ. The application of hemostatic cotton caused a pronounced lateral extrusion of the soft tissues, allowing a very good reproduction of the preparation limit. Moreover, the construction software allows the automatic definition of the preparation limit. In the next step, data acquisition of the opposite jaw and a digital bite registration by lateral scanning were performed.

**Figure 7:**
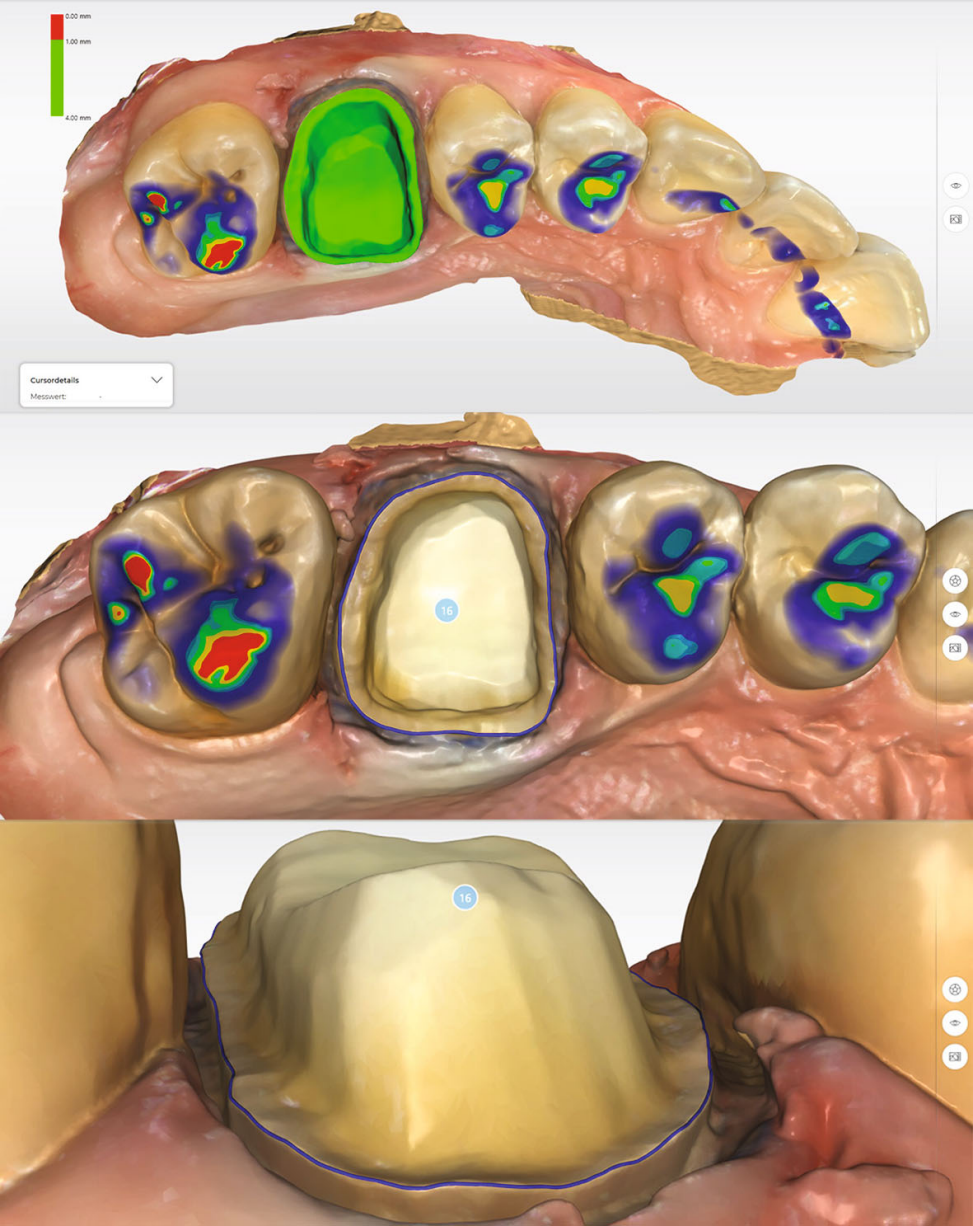
Analysis of preparation and determination of the preparation limit. The entire preparation limit is shown and can be detected automatically.

**Figure 8:**
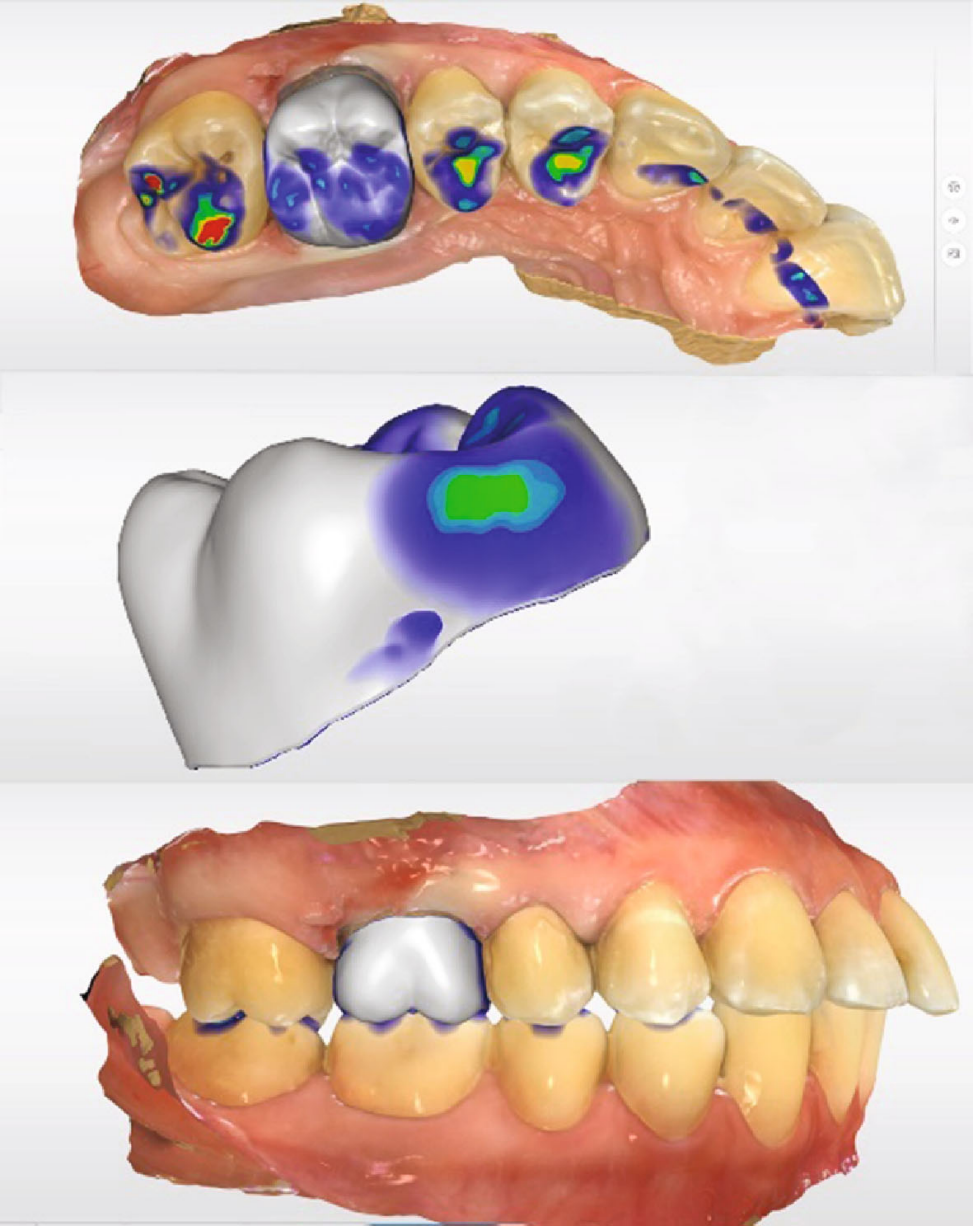
Virtual crown design (CEREC SW 5.2.2., Dentsply Sirona, Charlotte, USA). The occlusal contact points are reduced/built-up until they are depicted in turquoise. The design of the proximal contacts should be optimized to show light green areas.

After the final data acquisition, the preparation was analyzed by construction software (CEREC SW 5.2.2, Dentsply Sirona, Charlotte, USA). The occlusal reduction was evaluated as sufficient, and no undercuts were detected (Figure 7). The automatically determined preparation limit could be transferred without modifications (Figure 7). A modification of the restoration design was only necessary by adjusting the occlusal and proximal contacts. As the zirconia restoration requires glazing during the fabrication process, the contacts should be designed as small areas (color of contact point: turquoise, Figure 8). The proximal contacts should be designed more distinct (color of contact point: light green, Figure 8), in order to ensure sufficient contact to the adjacent teeth. If the contacts are too tight, they can be easily adjusted with a diamond-coated polishing lens. A too weak proximal contact point would require comparatively more time for correction, as a new glaze firing is required in this case (Table 1).

**Figure 9:**
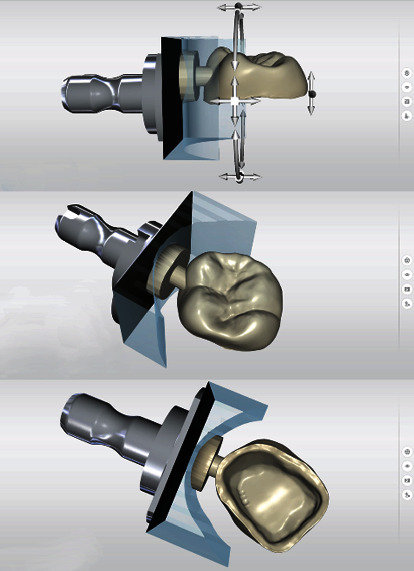
Vertical positioning of the restoration to determine the desired color gradient and the fixation area of the restoration.

In the next step, the vertical position of the restoration within the CEREC MTL Zirconia block was determined. Normally, the restoration is placed in the middle of the block, thus covering the entire color gradient [23, 29, 30]. In the present case, the adjacent teeth showed a pronounced portion of whitish enamel. In order to achieve a distinct visible incisal part, the restoration was placed approximately 1 mm below the surface of the block (Figure 9).

The crown was fabricated in a dry-milling process (Primemill, Dentsply Sirona, Charlotte, USA) from a multilayer translucent zirconia block (CEREC MTL Zirconia mono, Dentsply Sirona, Charlotte, USA). For fabrication, the milling mode “fine” was selected and the settings for the occlusal and radial spacer were 120 *μ*m (standard settings).

The restoration was milled from a block in color A3. This selection was based on the basic shade of the buccal surfaces of the adjacent teeth.

**Figure 10:**
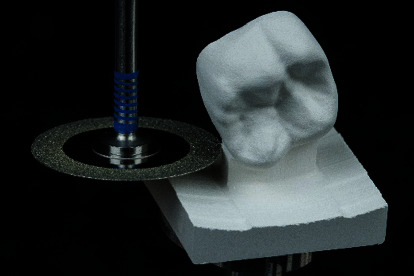
Milled restoration made of CEREC MTL Zirconia (Dentsply Sirona, Charlotte, USA). A diamond-coated separating disk is used to remove the retention pin.

Apart from the mode “fine,” a “superfast” milling mode is available. When using the superfast mode, a zirconia restoration can be dry-milled in 5 to 6 minutes. If this mode is chosen, it is essential to set the minimum material thickness parameter from 0.6 to 0.7 mm before processing. After milling, the retention pin of the crown was removed, and the connecting area was finished with a fine-grid diamond instrument (Figure 10).

Afterwards, the crown was densely sintered in an 18-minute speed-sintering process (CERECSpeedfire, Dentsply Sirona, Charlotte, USA). The CEREC software transferred all the information required for this process directly to the sintering unit (Table 1). If required, a try-in of the restoration is possible at this stage in order to check the occlusal and proximal contacts. The authors' experience shows that only corrections of the proximal contacts are required if the contact points are adjusted during the design process as described. These adjustments can be made after the final glazing of the crown without compromising the esthetic results by using a diamond-impregnated polyurethane polishing instrument for high-strength ceramics (e.g., 94013C, Komet Dental, Lemgo, Germany). Therefore, in the present case, the authors forwent a try-in of the restoration and directly started the color characterization.

**Figure 11:**
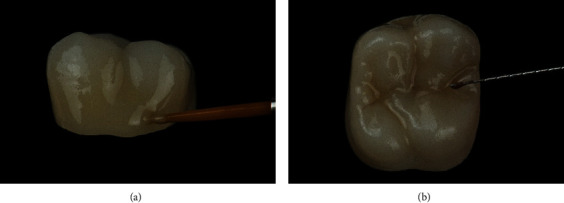
(a) Individualization of the cervical part of the restoration (Dentsply Sirona Universal Stain, color: Body S1, Dentsply Sirona, Charlotte, USA). (b) Fissure characterization with Dentsply Sirona Universal Stain, color Mahogany. A manual endodontic instrument (ISO 015) is ideal for precise application.

For this step, the entire external surface of the crown was covered with a transparent glazing (DS Universal Overglaze, Dentsply Sirona, Charlotte, USA). A layer of effect ceramic was applied to intensify the coloring of the cervical parts of the restoration (DS Universal Body Stain S1, Dentsply Sirona, Charlotte, USA) (Figure 11(a)). Finally, the fissures were color-characterized with a staining liquid (DS Universal Stain Mahogany, Dentsply Sirona, Charlotte, USA). To achieve the best results in coloring, a fine root canal instrument (e.g., reamers, ISO 010) is fit (Figure 11(b)). Finally, the cusp summits and cusp slopes were individualized. In the present case, the whitish parts of the adjacent teeth had to be mocked by a suitable staining liquid (DS Universal Incisal Stain I1, Dentsply Sirona, Charlotte, USA). After color application, the combined glaze-stain firing was performed at 760°C with a holding time of 2 minutes. The total processing time of the glazing was 9 minutes. Finally, the restoration was high-shine polished with a diamond polishing paste (Dura-Polish Dia, Shofu Inc., Kyoto, Japan).

**Figure 12:**
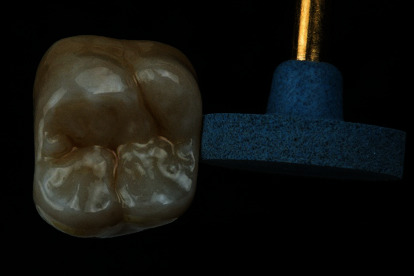
Adjustment of proximal contact points with a diamond-coated polyurethane polishing instrument.

In most cases, an esthetically optimized restoration can be fabricated with a colorless glazing material and 3 staining colors only (DS Body Stain S1, DS Incisal Stain S1, DS Mahogany, Dentsply Sirona, Charlotte, USA) in a single firing process, thus resulting in significantly shorter processing times. Afterwards, the individualized crown was tried in. An occlusal adjustment was not necessary. Only the proximal contacts were a little too tight; they were adjusted with a 2-step polishing system for high-strength ceramics. The contact point was slightly reduced with a prepolishing instrument (94020 C, Komet Dental, Lemgo, Germany) (Figure 12). In a second step, the revised areas were polished with a suitable instrument (94020F, Komet Dental, Lemgo, Germany).

**Figure 13:**
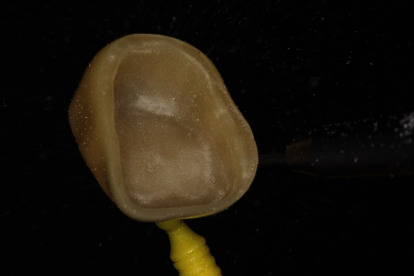
Prior to cementation, the internal surface of the crown is air-abraded with fine-particle-size alumina (<50 *μ*m) at a pressure of 0.1–0.2 MPa.

**Figure 14:**
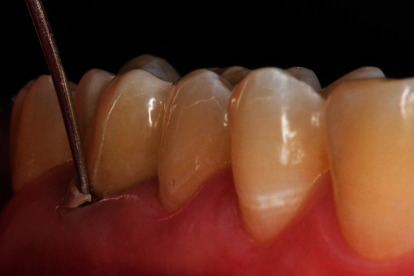
The comparatively easy removal of excess cement after a short-time light polymerization (2-3 seconds) is a clinically relevant advantage of self-adhesive cements.

**Figure 15:**
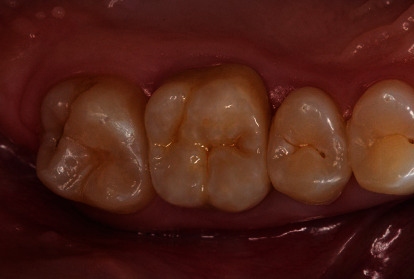
Occlusal view of the cemented monolithic zirconia crown.

**Figure 16:**
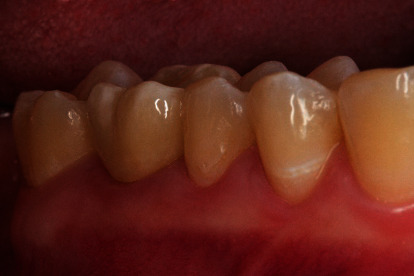
Lateral view of the final restoration 2 weeks after final insertion.

The restoration was conditioned for the final cementation by air-abrading with fine-grained alumina (50 *μ*m) at 0.1-0.2 MPa (Figure 13) and luted with a self-adhesive cement (Calibra Universal translucent, Dentsply Sirona, Charlotte, USA). After a short prehardening of the excess cement (2-3 seconds per surface), the excess material was removed with a probe or a scaler (Figure 14). The final light-curing was performed for 40 seconds each, buccal, occlusal, and lingual. The restoration proved to be a good color match with the adjacent teeth, and the patient was very satisfied with the esthetic results of the treatment (Figures 15 and 16).

## 3. Discussion

This case report describes the chairside fabrication of a monolithic zirconia crown. This type of restoration can generally be fabricated in a single appointment. However, it should be considered that this process is related to some technical preconditions (superfast milling, speed-sintering process, and multilayer zirconia material) [1, 2, 8, 9].

The use of a speed-sintering process is an important technical precondition. In the present case report, a special sintering oven for the CEREC system (CERECSpeedfire, Dentsply Sirona, Charlotte, USA) was used [2, 8]. Zirconia materials can only be speed-sintered while they are dry. This requires new equipment that allows for dry processing, e.g., the newer CEREC milling and grinding units (e.g., CERECPrimemill, Dentsply Sirona, Charlotte, USA). If zirconia is grinded in a wet state, a 20-minute drying process is required before sintering [8, 9]. This prolongation is considered counterproductive in the chairside fabrication of dental restorations. The “superfast” mode of the CERECPrimemill leads to a significant reduction in grinding time. In this mode, a full crown restoration can be fabricated in 5-6 minutes [16, 23].

It has to be considered that speed-sintering is a relatively new processing technique. Several in vitro studies have demonstrated that for suitable zirconia materials, this cost- and time-effective processing technique has no negative influence on the mechanical and optical properties such a hardness, fracture toughness, Weibull characteristics, hydrothermal aging behavior, and translucencies [8, 9, 24, 27, 28]. Nevertheless, up to date some possible aspects on clinical relevant factors (e.g., biocompatibility) remain unclear [26, 30]. Using a multilayer zirconia material offers the advantage that the typical color gradient in teeth, from the cervix to the incisal area, is already integrated with the finally sintered restoration. This specific precoloring allows for an esthetically satisfying individualization in a combined stain and glaze firing—this is an essential feature in the chairside fabrication of dental restorations because it leads to a reduced processing time compared to single-color zirconia materials [13, 14, 26].

Due to the constant development of the design software, the current version (CEREC SW 5.2.2, Dentsply Sirona, Charlotte, USA) offers practical design proposals that mainly only need minor adjustments at the occlusal and proximal contact points [14]. This adds to the significant reduction in design process times [16].

With the procedures and materials presented in this case report, a monolithic zirconia crown can technically be fabricated in approximately 75-90 minutes. Compared to previous fabrication processes, this is a significant shortening of chairside processing times for zirconia restorations [16]. However, independent from the fabrication procedure, the individual aspects of material choice have to be considered. Above all, this applies to the color choice of the zirconia block. When using zirconia of the fourth generation, the color of the restoration might be influenced by the color of the prepared stump. As the translucency of the material increases with decreasing layer thickness, the risk of a shining-through of the stump must be addressed, especially in the cervical area. High-translucent ceramic materials have limited applicability for the restoration of discolored stumps [2, 17, 21, 26].

For optimal performance in molar restorations, it is helpful to choose the block color (CEREC MTL Zirconia) one shade darker than the tooth color if the restoration has a wall thickness of approximately 1 mm. This is based on the clinical experience that the sintered restorations were too light when the determined target shade was used. Although color adaptation was possible, it required a second stain firing. In contrast, when choosing a block in a darker shade, the correct color match could be obtained in one combined stain and glaze [2, 21, 23]. However, this standard of choosing a color should not be applied in restorations with thick walls, as the crowns appear darker with an increased wall thickness [2, 21]. In these cases, the restoration should be milled from the color that was determined to be the target shade.

According to the manufacturer, the minimum material thickness of multilayer zirconia of the fourth generation can be reduced to 0.6-0.7 mm due to the comparably high flexural strength (>850 MPa) compared to glass-ceramic materials. This would present a major advantage of this new material, as the minimum required wall thickness allows for a minimum-invasive preparation. Moreover, the strength of the material offers the option of temporary cementation, which is not possible for high-strength glass-ceramics and zirconia of the third generation [6, 12, 16].

Nevertheless, as it happens with other restorative esthetic materials, also the flexural resistance of zirconia frameworks could be altered by several factors over time (e.g., acidic environment or cyclic load) [33, 34]. These variables should be evaluated in future tests before a general recommendation of these reduced material thicknesses can be given.

Moreover, it is necessary to mention that the fabrication process presented in this report is based on comprehensive in vitro data [1, 16, 20, 21], whereas results from clinical studies are still not available. This represents a limitation of the present case report. Therefore, the manufacturer's instructions, especially regarding the indication range, the recommended preparation design, cementation, and technical processing parameters, must be carefully observed [2]. An alternative treatment option for the case presented would have been the chairside fabrication of a crown made from a high-strength glass-ceramics (e.g., IPS e.max CAD, Ivoclar Vivadent, Schaan, and Liechtenstein). This treatment alternative has the advantage of less prerequisites regarding the milling process and the postprocessing as no dry-milling or speed-sintering is necessary. On the other hand, the minimum material thickness for glass-ceramic restorations is slightly higher than for the multilayer zirconia, and a temporary luting of these restorations is not possible.

## 4. Conclusions

Overall, if the technical prerequisites (superfast dry-milling and speed-sintering processes) are given, the fabrication of a zirconia restoration can easily be performed in a dental practice. The use of a multilayer zirconia block of the fourth generation facilitates the process and allows for a time-saving esthetic individualization of the restoration.

## Figures and Tables

**Table 1:** Clinical and technical procedures (step-by-step) for the chairside fabrication of multilayer zirconia crowns.

Step	Clinical procedures	Technical procedures
1	Adhesive build-up Preparation Soft tissue management Placement of retraction cord	
2	Intraoral scanning Preparation, antagonistic jaw and buccal scan (bite registration)	
3		CAD design Preparation analysis (substance reduction, undercuts, artefacts) Determination of preparation limit Modification of the restoration design
4		CAM process Selection of ceramic block Dry-milling Speed-sintering
5		Staining and glazing of the restoration Conditioning of the intaglio restoration surface (air-abrading process)
6	Try-in Verification of correct shade Occlusal and proximal adjustments if needed	
7	Luting Definitive cementation (e.g., self-adhesive cement) or provisional cementation	
